# Spindle cell oncocytoma of the adenohypophysis: Two case reports and a review of the literature

**DOI:** 10.3892/mmr.2015.3476

**Published:** 2015-03-12

**Authors:** QINGCHUN MU, JINLU YU, LIMEI QU, XITONG HU, HAIJUN GAO, PENGFEI LIU, XU ZHENG, YUXUE SUN, HAIYAN HUANG

**Affiliations:** 1 Department of Neurosurgery, The First Hospital of Jilin University, Changchun, Jilin 130021, P.R. China; 2 Department of Neurosurgery, Hongqi Hospital, Mudanjiang Medical University, Mudanjiang, Heilongjiang 157011, P.R. China; 3 Department of Pathology, The First Hospital of Jilin University, Changchun, Jilin 130021, P.R. China

**Keywords:** spindle cell oncocytoma, surgical resection, pathological diagnosis

## Abstract

Spindle cell oncocytoma (SCO) of the adenohy-pophysis is a rare tumor in the sellar region. Due to its rarity, little information is available regarding SCO. It is often misdiagnosed as another type of sellar tumor. In the present study, two cases of SCO were reported. One patient was a 35-year-old female presenting with decreased visual acuity, amenorrhea and lactation. The other patient was a 62-year-old female with no clear symptoms or signs. Cranial magnetic resonance imaging (MRI) revealed a suprasellar mass with marked homogeneous enhancement in the two cases. A craniotomy was performed to completely resect the tumors. The tumors were immunopositive for vimentin, epithelial membrane antigen (EMA), S-100 and thyroid transcription factor-1 (TTF-1). The tumors were pathologically diagnosed as SCO. No recurrence occurred during the follow-up period of 15–21 months. In the present study, the literature was reviewed and the clinical data, imaging features, intraoperative findings and recurrence of 24 cases were analyzed in the literature as well as the present two cases. The average age of the SCO patients was 58.5 years and no gender preference was observed for the disease. The tumor exhibited homogeneous enhancement on the MRI. The intraoperative assessment revealed that the tumor had a rich blood supply and the SCO tumors were immunopositive for vimentin, S-100, EMA and TTF-1. These findings provided valuable clinical data for the preoperative diagnosis and surgical removal of SCO tumors.

## Introduction

Spindle cell oncocytoma (SCO) of the adenohypophysis is a rare benign tumor in the sellar region, accounting for 0.1–0.4% of all sellar tumors (1,2). SCO was first identified as a distinct entity by the World Health Organization (WHO) classification of tumors of the central nervous system (CNS) in 2007 (3). To date, only 24 cases have been reported in the literature (1,2,4–18). Due to its rarity, little information is available regarding the imaging features and surgical characteristics of SCO (2). As SCO shares a similar clinical presentation and imaging features with nonfunctional pituitary adenoma, it is often misdiagnosed as a nonfunctional pituitary adenoma (7). However, SCO tumors have a greater blood supply than pituitary adenomas, thereby increasing difficulties associated with surgical removal of the tumor (15). Preoperative misdiagnosis as pituitary adenoma may result in an underestimation of the surgical difficulty. In the present study, two cases of SCO were reported and 24 cases of SCO in the literature were reviewed. The imaging features, intraoperative findings, immunohistochemical features and prognosis of SCO were summarized. The present study provided important clinical information for the correct preoperative diagnosis and intraoperative removal of SCO tumors. Written informed consent was obtained from all of the patients.

## Case report

### Case 1

**Figure 1:**
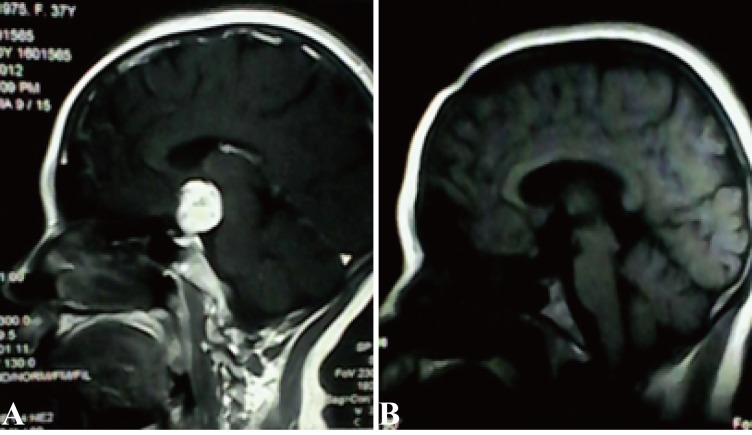
MRI images of case 1. (A) Preoperative MRI image revealing a suprasellar round mass. (B) MRI image at the 21-month follow-up exhibiting no recurrence of the tumor. MRI, magnetic resonance imaging.

A 35-year-old female was admitted to the First Hospital of Jilin University (Changchun, China), who had been presenting with amenorrhea and lactation for two years. On examination, her visual acuity was 0.8 in the left eye and 0.5 in the right eye. Dark spots were observed in the left inferior temporal quadrant and decreased light sensitivity was observed in the right nasal quadrant. Cranial magnetic resonance imaging (MRI; 3.0 Tesla Trio MRI Scanner; Siemens AG, Erfurt, Germany) revealed a suprasellar round mass of 2.5×3.0×1.0 cm, with equal T1 and T2 signals. This mass exhibited marked homogeneous enhancement (Fig. 1A). Laboratory assessments used to examine pituitary disorders revealed an elevated level of prolactin (34.62 ng/ml; normal range, 1.4–24.0 ng/ml), a decreased level of luteinizing hormone (LH; 1.410 IU/l; normal range, 2.12–10.891 IU/l) and normal levels of follicle-stimulating hormone (FSH), corticotropin and thyrotropin.

The patient was diagnosed as having a pituitary prolactin adenoma. The tumor was resected through the second gap using a right expanded frontotemporal craniotomy. The tumor was gray with blood vessels on the surface, was firm without clear encapsulation and had an appearance similar to that of normal brain tissue with a rich blood supply. The pituitary stalk was adherent to the tumor and was pushed inferiorly by the tumor. The stalk was partially preserved following careful dissection. The tumor was removed section by section until complete removal of the tumor was achieved by visualization under a microscope. Persistent diabetes insipidus occurred following surgery and it was gradually relieved following oral administration of desmopressin acetate for 2.5 months. An enhanced MRI performed at seven days after surgery confirmed the complete removal of the tumor. No recurrence had occurred by the time-point of the 21-month follow-up examination (Fig. 1B).

**Figure 2:**
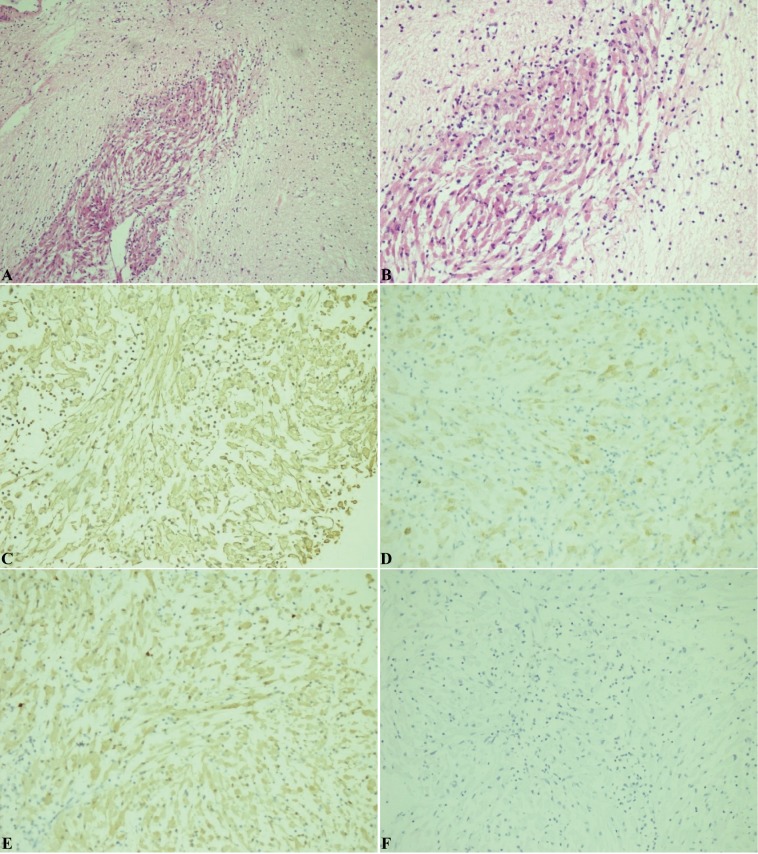
Histological and immunohistochemical staining of the spindle cell oncocytoma in case 1. (A and B) Hematoxylin and eosin staining revealed that the tumor was composed of spindled and epithelioid cells with an abundant eosinophilic cytoplasm. Infiltration of scattered mature lymphocytes was observed in the extracellular matrix. A, magnification, ×100; B, magnification, ×200. Immunohistochemical staining for (C) vimentin, (D) epithelial membrane antigen, (E) S-100 and (F) glial fibrillary acidic protein. C-F, magnification, ×20.

Postoperative hematoxylin and eosin (H&E)-stained sections revealed that the tumor was composed of spindled and epithelioid cells arranged in nests and sheets. The cells had an abundant eosinophilic cytoplasm. Mild to moderate nuclear atypia was identified; however, mitosis was not observed. Nuclear pleomorphism was observed in certain cells and a double nucleus was occasionally present. Infiltration of scattered mature lymphocytes was observed in the extracellular matrix (Fig. 2A and B). The tumor was immunonegative for GFAP and Syn, whilst it was immunopositive for vimentin, EMA, S-100 and TTF-1 (Fig. 2C-F). The mindbomb E3 ubiquitin protein ligase 1 (MIB-1) labeling index was ~3%. The tumor was pathologically diagnosed as SCO.

### Case 2

**Figure 3:**
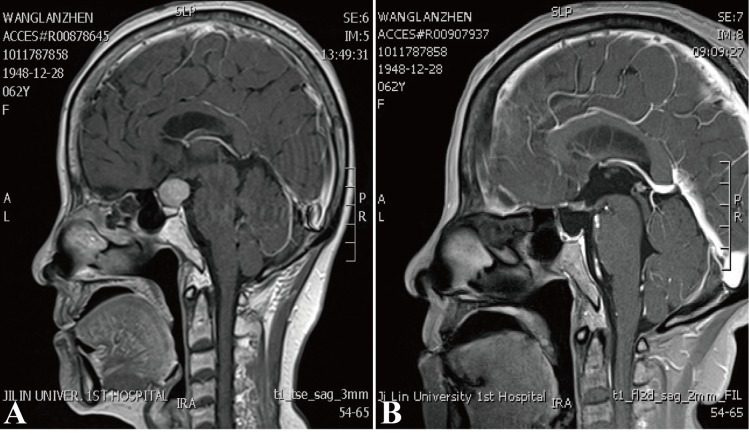
MRI images of case 2. (A) Preoperative MRI image demonstrating a suprasellar mass. (B) MRI image at the 15-month follow-up revealing no recurrence of the tumor. MRI, magnetic resonance imaging.

A 62-year-old female was admitted to the First Hospital of Jilin University due to a sellar mass identified during a routine physical examination. She presented no clear symptoms or signs. Cranial MRI revealed a suprasellar mass of 2.3×1.7×2.0 cm, with long T1 and short T2 signals (Fig. 3A). This mass exhibited marked homogeneous enhancement. The pituitary gland was flattened due to tumor compression and the pituitary stalk was not clearly observed. The optic chiasm was elevated; however, the cavernous sinus was not invaded by the tumor. Laboratory assessments used to examine pituitary disorders revealed a decreased level of LH (0.390 IU/l; normal range, 2.12–10.891 IU/l), but normal levels of pituitary hormones, including FSH, prolactin, corticotropin and thyrotropin.

The patient was diagnosed as having a nonfunctional adenoma. The tumor was resected between the first and second gap using a right transpterional craniotomy. The tumor was light yellow and slightly soft, and it had a brain stem-like appearance with a rich blood supply. The tumor was removed section by section. Care was taken to preserve the membrane-like pituitary stalk dorsolateral to the tumor until the tumor was completely removed. Transient diabetes insipidus occurred immediately following surgery; however, it was effectively treated following two weeks of oral administration of desmopressin acetate. An enhanced MRI performed at three days after surgery confirmed the complete removal of the tumor. No recurrence had occurred by the time-point of the 15-month follow-up examination (Fig. 3B).

**Figure 4:**
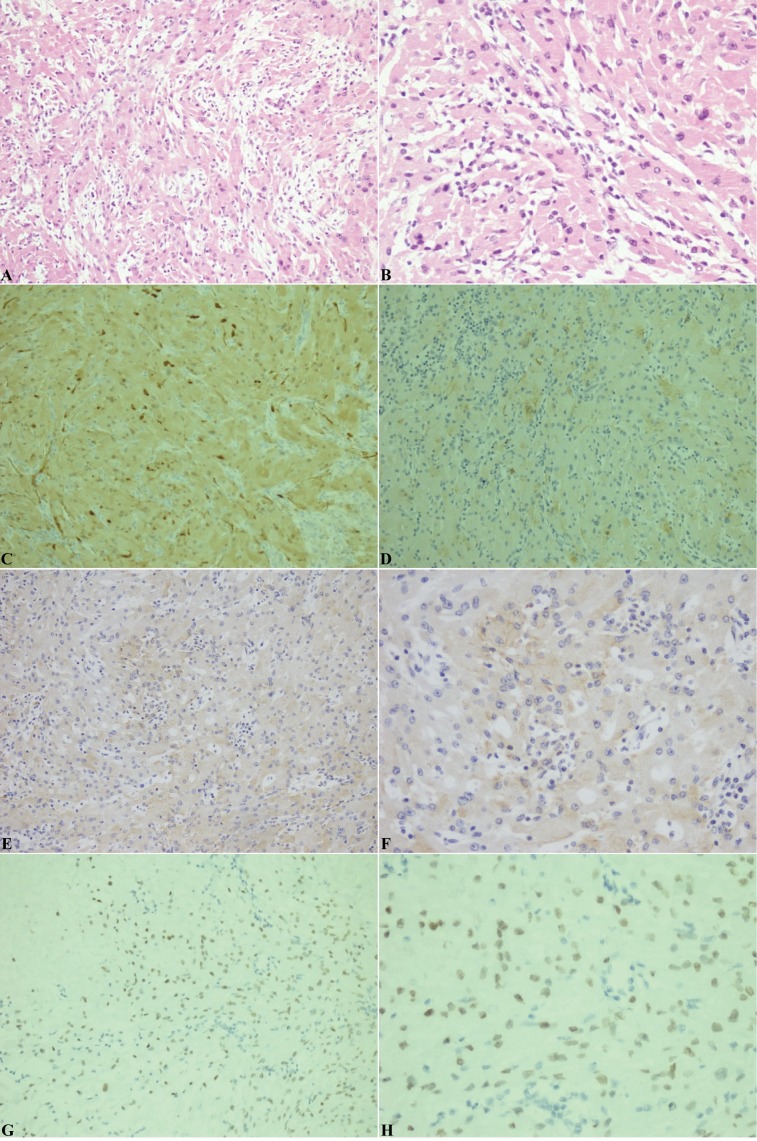
Histological and immunohistochemical staining of the spindle cell oncocytoma in case 2. (A and B) Hematoxylin and eosin staining revealing that the tumor was composed of spindled and epithelioid cells arranged in intersecting fascicles. The cells had an abundant eosinophilic cytoplasm. Mild to moderate nuclear atypia was identified, but mitosis was not observed. Nuclear pleomorphism was found in certain cells. Infiltration of a few mature lymphocytes and local interstitial mucoid degeneration was observed. A, magnification, ×20; B, magnification, ×40. Immunohistochemical staining for (C) S-100, (D) epithelial membrane antigen, (E and F) vimentin and (G and H) thyroid transcription factor-1. C-E and G, magnification, ×20; F and H, magnification, ×40.

Post-operative H&E-stained sections demonstrated that the tumor was composed of spindled and epithelioid cells arranged in intersecting fascicles. The cells had an abundant eosinophilic cytoplasm with round or oval nuclei and inconspicuous nucleoli. Mild to moderate nuclear atypia was identified; however; mitosis was not observed. Nuclear pleomorphism was observed in certain cells. Infiltration of a few mature lymphocytes and local interstitial mucoid degeneration was observed (Fig. 4A and B). The tumor was immunonegative for GFAP, creatine kinase, Syn and B-cell lymphoma 2; however, it was immunopositive for vimentin, EMA, S-100 and TTF-1 (Fig. 4C-F). The MIB-1 labeling index was ~1.5%. The tumor was pathologically diagnosed as SCO.

## Discussion

SCO was first reported by Roncaroli *et al* (1) in 2002 in five patients and it was later identified as a novel entity by the WHO classification of tumors of the CNS in 2007 (3). Histologically, SCO cells are mainly composed of a bundle of spindle cells with an eosinophilic and granular cytoplasm. They are immunopositive for vimentin, EMA, S-100 and galectin-3, but immunonegative for pituitary hormones, chromogranin and Syn. SCO is similar to nonfunctional pituitary adenoma and accounts for 0.1–0.4% of all sellar tumors (1,2). In a retrospective study of 2,000 cases of pituitary tumors, only two cases were diagnosed as being SCO (2). Due to its rarity, only 16 studies were available describing 24 cases of SCO using a PUBMED search (http://www.ncbi.nlm.nih.gov/pubmed) for studies published between 2002 and 2013. The clinical characteristics, intraoperative findings as well as the imaging and immunohistochemical features of the 24 published cases, and the two cases reported in the present study were assessed. SCO commonly occurred in middle-aged and elderly males or females. Of the 24 cases published in the literature, 10 patients were male and 14 were female. No gender preference for SCO is therefore present. The average age of the SCO patients was 56.4 years (range, 24–76 years). In the present two cases, the patients were females aged 35 and 62 years old, respectively.

Similar to nonfunctional pituitary adenoma, the most common clinical manifestations of SCO are visual impairment and panhypopituitarism. Of the 24 cases in the literature, 14 cases presented with decreased or impaired visual acuity and 12 cases presented with panhypopituitarism. In addition, intermittent epistaxis was reported in one patient with a large SCO (4), two cases exhibited weight loss (2,5) and one case had long-term musculoskeletal pain (6). A total of three cases had decreased libido or sexual dysfunction (7–9) and two cases presented with oligomenorrhea or amenorrhea (10,11). Consistent with previous studies, one patient (case 1) in the present study presented with decreased visual acuity, amen-orrhea and lactation. However, the other patient (case 2) did not exhibit any clear symptoms or signs. Furthermore, postoperative panhypopituitarism and diabetes insipidus occurred in four cases (12–15). One case required hormone replacement therapy for 15 years (12). In the present two cases, postoperative diabetes insipidus occurred and was treated following oral administration of desmopressin acetate for two weeks and two months, respectively. Therefore, similar to other sellar tumors, SCO often leads to pituitary hormone disorders and is associated with postoperative complications, such as diabetes insipidus.

SCO is often misdiagnosed preoperatively. Of the 24 cases of SCO in the literature, 14 cases were misdiagnosed as being nonfunctional adenomas. Due to its spindle-like shape, four cases were misdiagnosed as being schwannomas. In addition, one case was misdiagnosed as being a craniopharyngioma due to recurrent intratumoral bleeding (13). Furthermore, two cases were misdiagnosed as being null cell pituitary adenomas (4). In the present two cases, one was misdiagnosed as being a nonfunctional adenoma and the other was misdiagnosed as being a pituitary prolactin adenoma.

SCO shares similar imaging features with nonfunctional adenoma, exhibiting no dural attachment or invasion (19). Of the 24 cases in the literature, the size and site of the tumor on MRI images were described in 23 cases. Suprasellar or intrasellar tumors were reported in 20 cases. Only two cases reported that the tumor was located within the sella turcica (10,11). Tumor invasion to the cavernous sinus and compression of the temporal lobe was reported in one case (9). In addition, one case reported that the tumor grew forward and invaded into the sphenoid, ethmoid, nasopharynx and posterior nasal cavity, leading to intermittent epistaxis (4). Borota *et al* (6) reported one case of SCO, which had invaded into the sphenoid sinuses and disrupted the body of the sphenoid bone, including the sella turcica. In the present two cases, the two tumors were large and suprasellar, without invasion into the cavernous sinuses. These findings are consistent with a meta-analysis of the world literature since 1893 by Covington *et al* (20), revealing that SCO is either suprasellar or intra- and supra-sellar. Computed tomography (CT) images of SCO were only reported in three cases (5,13,16). Borges *et al* (13) reported that ~50% of SCO tissues exhibited calcification on CT images, consistent with the local hyperintense signals on T1-weighted MRI. In addition, Singh *et al* (16) demonstrated that SCO exhibited isointensity to the cerebral parenchyma on CT images without intratumoral calcification or bleeding. In addition, as SCO commonly has a rich blood supply, the tumor may exhibit enhancement on MRI. In the present cases, the two SCO tumors exhibited marked homogeneous enhancement. Similarly, Fujisawa *et al* (15) reported that SCO exhibited numerous and faint intratumoral vessels on a magnetic resonance angiogram (MRA); angiography revealed that the SCO was extensively fed by the bilateral internal carotid arteries and draining veins were observed in the arterial phase (15). Of five cases of SCO with a rich blood supply as determined by a preoperative MRA, severe intraoperative bleeding occurred in four cases (6,8,9,15). However, as SCO was misdiagnosed as nonfunctional adenoma preoperatively, angiography was not performed in the majority of cases of SCO. Therefore, preoperative angiography should be performed to evaluate the blood supply of SCO if a rich blood supply is suspected on the MRA, thereby reducing the risk of intraoperative bleeding.

Of the 24 cases of SCO in the literature, 13 cases described the intraoperative findings. The SCO was described to be pale gray (10), grayish gelatinous (7) and yellow (11). Similarly, the SCO was gray or yellow in the present two cases. The texture was similar to that of normal brain tissue with a rich blood supply. In addition, 11 cases described the SCO as a firm and vascular tumor. Kloub *et al* (4) reported that two recurrent SCOs were invasive with an unclear boundary with the surrounding tissues and necrosis was identified in one tumor. Tumor invasion into the base of the sella turcica occurred in two cases (4,9). No tumor invasion was reported via intraop-erative inspection in any of the other cases. Dahiya *et al* (9) reported a case of SCO that was firm and difficult to dissect and residual tumors were found around the internal carotid artery following the second surgical procedure. Intratumoral bleeding was reported in two cases and tumors with a rich blood supply were reported in seven cases. Severe intraopera-tive blood loss (600–900 ml) was also reported (13,15).

SCO tumors are immunopositive for vimentin, S-100 and EMA (1,2,4–6,10–12,14,15,16). Electron microscopy revealed that SCO cells contain abundant swollen mitochondria and that bundles of intermediate filaments are entrapped in the lysosomes and profiles of the rough endoplasmic reticulum (1). Roncaroli *et al* (1) and Borges *et al* (13) reported that well-formed desmosomes and intermediate junctions, but not secretory granules, were observed in SCO cells. However, several other studies revealed that occasional electron dense secretory granules, but not desmosomes or intercellular junctions, were observed in SCO cells (4,9,16). Based on the immunohistochemical and ultrastructural similarities shared by SCO and folliculostellate cells (FSCs), SCO is theorized to originate from FSCs (1,21,22). FSCs are star-like nonhor-mone-secreting cells in the anterior pituitary, which provide structural support for hormone-secreting cells, accounting for 5–6% of the pituitary cell population (23,24). FSCs are hypothesized to be adult stem cell-like pituitary cells, which have a capacity for divergent differentiation (4).

Lee *et al* (25) described the expression of TTF-1 in eight cases of SCO and demonstrated that TTF-1 was generally expressed in the fetal neurohypophysis. Similarly, Mlika *et al* (14) reported one case of SCO with positive TTF-1 expression. In the present study, positive TTF-1 expression was identified in the two cases of SCO. These findings suggested that this marker may be specific to human pituicytes. The positive expression of TTF-1 in these 11 cases of SCO may facilitate further studies on the classification of these rare sellar tumors and may suggest that SCO and pituicytoma have a similar origin (25). In addition, Mete *et al* (26) reported positive TTF-1 expression in seven cases of SCO, four cases of pituicytomas and three cases of granular cell tumors of the pituitary; while all cases were negative for FSCs. The authors hypothesized that SCO and granular cell tumors are variants of pituicytoma and proposed the terms ‘oncocytic pituicytoma’ and ‘granular cell pituicytoma’ to refine the classification of these lesions (26). Alexandrescu *et al* (11) observed that SCO was positive for CD44, nestin and SMI-131, suggesting that SCO has features that are similar to those of neuronal precursors, which may explain the recurrence of SCO in certain cases.

Of the 24 cases of SCO in the literature, recurrence occurred in eight cases with an average recurrence time of 3.3 years (range, 5–13 years). The mean MIB-1 labeling index was 3%. A total of two cases with a high MIB-1 labeling index (10–20%) exhibited recurrence. The other six cases with a low MIB-1 labeling index also exhibited recurrence. Of the six cases treated with radiotherapy (doses of 50–55 Gy), recurrence occurred in four cases. No intra- or extracranial metastases were identified. However, the longest follow-up period was 16 years (12). These findings suggested that SCO patients should be followed up for five years or more and it may not be appropriate to define SCO as a WHO grade I tumor with a short follow-up period. In addition, Ogiwara *et al* (7) found that an incomplete resection of the tumor was a significant risk factor for the recurrence of SCO. Therefore, a complete resection of the tumor is necessary to prevent the recurrence of SCO.

In conclusion, SCO was identified as a novel type of tumor in the WHO classification of tumors of the CNS in 2007 (3). However, to date, only 24 cases of SCO have been reported in the literature and little information regarding SCO is available. Similar to nonfunctional adenoma, the most common clinical manifestation of SCO is panhypopituitarism. Tumors with an enhancement on an MRI should be considered as SCO and MRA and/or angiography should be performed to assess the blood supply of the tumor, thus preventing the risk of severe intraoperative bleeding. Complete removal of the tumor is important to prevent tumor recurrence. The intraoperative findings, including the texture and the blood supply of the tumor, may provide valuable clinical information to guide the surgical procedures.
